# Overcoming Temozolomide Resistance in Glioblastoma via Enhanced NAD^+^ Bioavailability and Inhibition of Poly-ADP-Ribose Glycohydrolase

**DOI:** 10.3390/cancers14153572

**Published:** 2022-07-22

**Authors:** Jianfeng Li, Christopher A. Koczor, Kate M. Saville, Faisal Hayat, Alison Beiser, Steven McClellan, Marie E. Migaud, Robert W. Sobol

**Affiliations:** 1 Department of Pharmacology, College of Medicine, University of South Alabama, Mobile, AL 36688, USA; jianfengli@southalabama.edu (J.L.); cakoczor@southalabama.edu (C.A.K.); mkm1325@jagmail.southalabama.edu (K.M.S.); fhayat@southalabama.edu (F.H.); avbeiser@southalabama.edu (A.B.); mmigaud@southalabama.edu (M.E.M.); 2 Mitchell Cancer Institute, University of South Alabama, Mobile, AL 36604, USA; 3 Mitchell Cancer Institute Flow Cytometry SRL, University of South Alabama, Mobile, AL 36604, USA; smcclellan@health.southalabama.edu

**Keywords:** glioblastoma, NAD^+^, PARG inhibition, PARP activation, temozolomide, MSH6, MGMT, BER, MMR, TMZ resistance

## Abstract

**Simple Summary:**

Glioblastoma is the most prevalent and lethal brain tumor type, often treated with the DNA alkylating agent temozolomide (TMZ). The cytotoxic DNA lesion O^6^-methylguanine only accounts for about 9% of the DNA lesions induced by TMZ. The other DNA lesions (>80%) are quickly repaired by the base excision repair (BER) pathway. However, resistance to cytotoxicity of the O^6^-methylguanine lesion is common in cancer due to defects in the mismatch repair pathway or overexpression of the MGMT repair protein. Therefore, the aim of this study was to find approaches to inhibit the BER pathway to overcome TMZ resistance. We found that combining TMZ with an NAD^+^ precursor (dihydronicotinamide riboside) and a PARG inhibitor strongly inhibited BER and overcame TMZ resistance. This combination treatment regimen provides a novel approach to consider for glioblastoma.

**Abstract:**

Glioblastoma multiforme (GBM) is an incurable brain cancer with an average survival of approximately 15 months. Temozolomide (TMZ) is a DNA alkylating agent for the treatment of GBM. However, at least 50% of the patients treated with TMZ show poor response, primarily due to elevated expression of the repair protein O^6^-methylguanine-DNA methyltransferase (MGMT) or due to defects in the mismatch repair (MMR) pathway. These resistance mechanisms are either somatic or arise in response to treatment, highlighting the need to uncover treatments to overcome resistance. We found that administration of the NAD^+^ precursor dihydronicotinamide riboside (NRH) to raise cellular NAD^+^ levels combined with PARG inhibition (PARGi) triggers hyperaccumulation of poly(ADP-ribose) (PAR), resulting from both DNA damage-induced and replication-stress-induced PARP1 activation. Here, we show that the NRH/PARGi combination enhances the cytotoxicity of TMZ. Specifically, NRH rapidly increases NAD^+^ levels in both TMZ-sensitive and TMZ-resistant GBM-derived cells and enhances the accumulation of PAR following TMZ treatment. Furthermore, NRH promotes hyperaccumulation of PAR in the presence of TMZ and PARGi. This combination strongly suppresses the cell growth of GBM cells depleted of MSH6 or cells expressing MGMT, suggesting that this regimen may improve the efficacy of TMZ to overcome treatment resistance in GBM.

## 1. Introduction

Standard of care in the treatment of glioblastoma multiforme (GBM) includes maximum tumor resection followed by radiotherapy and multiple cycles of adjuvant temozolomide (TMZ, Temodar) [1,2]. However, somatic or acquired resistance to TMZ compromises its efficacy, with a median survival of GBM patients between 12 and 15 months [3,4]. TMZ is a DNA alkylating agent generating multiple DNA lesions, including N7-methylguanine (N7-MeG, >70%), N3-methyladenine (N3-MeA, ~10%), and O^6^-methylguanine (O^6^-MeG, ~9%) [5,6]. The cytotoxicity of TMZ is mainly derived from the O^6^-MeG lesion and a functional MMR pathway [7,8,9]. However, TMZ resistance is seen in tumor cells expressing the direct repair protein O^6^-methylguanine-DNA methyltransferase (MGMT) [10,11]. At least 50% of those treated with TMZ do not respond due to elevated expression of MGMT [6,12,13]. Another important factor that leads to TMZ resistance is a deficiency or mutation in one or more of the proteins of the mismatch repair pathway (MMR) since a functional MMR pathway is needed for the cytotoxicity of O^6^-MeG [14,15,16]. Current clinical- or pre-clinical-based studies point out that acquired resistance to TMZ primarily arises from the loss of MMR proteins such as MutS Homolog 6 (MSH6) or by gaining expression of MGMT [11,17,18].

Strategies that promote MGMT inhibition or BER inhibition are promising approaches to overcoming TMZ resistance [6,19]. However, a clinical trial testing a combination of Lomeguatrib, an MGMT inhibitor, with TMZ did not show any benefit from MGMT inhibition [20]. Combining a BER inhibitor (PARP inhibitor) with TMZ was found to induce G2/M arrest, an increase in double-strand DNA breaks, and elevated apoptosis in MSH6-deficient, TMZ-resistant glioblastoma cells [21]. However, a PARP1/2 inhibitor, CEP-9722, combined with TMZ only reported limited clinical efficacy [22]. A recent clinical trial with the addition of veliparib (ABT-888) to radiation followed by TMZ concluded that veliparib was tolerated but did not improve survival for patients with newly diagnosed diffuse intrinsic pontine glioma (DIPG) [23]. Therefore, new targets are needed for the potentiation of TMZ cytotoxicity. One promising target is poly(ADP-ribose) glycohydrolase (PARG), an important component of the BER pathway as the primary hydrolase responsible for the degradation of poly(ADP-ribose) (PAR) after the recruitment of DNA repair factors [24]. Genetic depletion of PARG or inhibition of PARG strongly increased the level of DNA damage induced by TMZ, methyl methanesulfonate (MMS), ionizing radiation (IR), or cisplatin and significantly reduced cell survival in different types of cancer cells [6,25,26,27,28,29]. We recently reported that supplementation with the NAD^+^ precursor dihydronicotinamide riboside (NRH) rapidly increased NAD^+^ levels in glioma stem cells (GSCs) and GBM cells, enhancing PARP1 activation. Further, we found that co-administration of NRH and a PARG inhibitor (PARGi) triggers hyperaccumulation of PAR, intra S-phase arrest, and apoptosis in GSCs but minimal PAR induction or cytotoxicity in normal astrocytes [25]. Therefore, in this study, we hypothesized that co-treatment of **T**MZ with **N**RH + **P**ARGi (T + N + P) would enhance the cytotoxicity of TMZ in TMZ-resistant GBM cells.

In the present report, we demonstrated that the administration of NRH together with TMZ and PARGi markedly potentiated the cytotoxicity of TMZ or TMZ + PARGi in TMZ-resistant cell lines such as those with a deficiency in MSH6 or due to elevated expression of MGMT. NRH treatment increased cellular NAD^+^ levels and promoted the accumulation of PAR from TMZ + PARGi treatment. Further, this treatment inhibited the activity of AKT (AKT serine/threonine kinase 1), an important survival factor for GBM. As a result, the growth of TMZ-resistant cells was strongly inhibited.

## 2. Materials and Methods

### 2.1. Cells and Cell Culture Conditions

LN428 and LNZ308 GBM cells were generous gifts from Dr. Ian Pollack (University of Pittsburgh). Gli60, an MSH6 mutant GBM cell line, was a gift from Dr. Daniel P. Cahill (Dana-Farber/Harvard Cancer Center, Massachusetts General Hospital). U87MG cells (Cat# HTB-14, ATCC), U2OS cells (Cat# HTB-96, ATCC), and T98G cells (Cat# CRL-1690, ATCC) were purchased from ATCC. 293-FT cells were purchased from Thermo Fisher Scientific (Cat# R70007, Waltham, MA, USA). LN428, LNZ308, U87MG, and T98G cells were cultured in MEM alpha (Cat#12571063, Thermo Fisher Scientific, Waltham, MA, USA) with 10% FBS(HI) and antibiotic/antimycotic (Cat#15240062, Thermo Fisher Scientific, Waltham, MA, USA). U2OS and Gli60 cells were cultured in DMEM (15-013-CV, Corning, Corning, NY, USA) with 10% FBS(HI), L-glutamine, and antibiotic/antimycotic. 293-FT cells were cultured in DMEM (Cat# 45000-304, VWR) with 10% FBS(HI), glutamine (2 mM) (Cat# 25030081, Thermo Fisher Scientific, Waltham, MA, USA), and antibiotic/antimycotic (Cat# 15240062, Thermo Fisher Scientific, Waltham, MA, USA). All cells were cultured at 37 °C with 5% CO_2_. The growth medium was changed every 3 or 4 days. Cells were treated with the following chemicals as described below and in the legends: PARG inhibitor (PDD00017273; Cat# SML1781, Sigma-Aldrich, St. Louis, MO, USA), TMZ (Cat# T2577, Sigma-Aldrich, St. Louis, MO, USA), or dihydronicotinamide riboside (NRH) [30].

### 2.2. Cell Extract for Immunoblot Analysis

Cells (1 × 10^6^) were seeded in a 100-mm dish and treated in 10 mL growth medium with different treatments for 24 h. Then, cells were collected and were lysed using 2× clear Laemmli buffer at a ratio of 100 μL Laemmli buffer per 10^6^ cells. Whole cell lysate (15 μL, ~30 μg) was used for immunoblotting analyses. The primary and secondary antibodies used are listed in Table 1. The intensity of each band was quantified using Image J [31].

### 2.3. Lentivirus Production and Cell Transduction

Four plasmids, including the packaging vectors (pMD2.g(VSVG), pVSV-REV and pMDLg/pRRE), and a shuttle vector (as indicated below) were transfected into 293-FT cells using the TransIT-X2 Transfection reagent (Cat# MIR6005, Mirus, Madison, WI, USA) to develop the corresponding lentiviral particles. The shuttle vector pLenti-U6-sgRNA(MSH6)-SFFV-Cas9-2A-Puro was used to generate lentivirus for the expression of Cas9 and gRNA specific to the human MSH6 gene, pLenti-CRISPR-cas9-Scramble for the expression of the Cas9/gRNA control (SCR-gRNA), pLV-CMV-XRCC1-EGFP-Hygro for the expression of XRCC1-EGFP, pLKO.1-puro-shSCR for the expression of the scrambled shRNA, or pLKO.1-puro-shMSH6.1 for the expression of MSH6-specific shRNA (see plasmid details in Supplementary Table S1). Viral particles were then isolated 48 h after transfection by filtering the supernatant through 0.45-μM filters, as described previously [25,32]. A Lenti-X Concentrator (Takara Bio, Cat# 631231, Kusatsu, Japan) was then used to concentrate the lentivirus particles, according to the manufacturer’s instructions.

### 2.4. MSH6 Knockdown by Expression of shRNA

The protein levels of MSH6 in LN428 cells were suppressed by stable expression of MSH6-specific shRNA (see the plasmid details in Supplementary Table S1). Briefly, LN428 cells were transduced with lentivirus expressing either a control (Scrambled, shSCR) shRNA or an MSH6-specific shRNA and selected in puromycin (1.0 µg/mL) for 2 weeks. Loss of MSH6 expression was then validated by immunoblot (see plasmid details in Supplementary Table S1).

### 2.5. MSH6 Knockout by CRISPR/Cas9

A set of three plasmids, with each gRNA targeting a different sequence of the MSH6 gene, was purchased from ABM (Cat# 3073611, Applied Biological Materials Inc., Richmond, VA, Canada). The gRNA targeting sequences are AAGGCGAAGAACCTCAACGG, GGGTGGTTGTAAACCAGACA, or ACAGTAGTCGCCCTACTGTT, specific for MSH6 (see the plasmid details in Supplementary Table S1). After the collection and concentration of the individual lentivirus particles, the three concentrated lentivirus particles were combined and then used to transduce the cells with the mixed lentivirus. This concentrated, high-titer virus mixture was able to completely deplete the expression of MSH6 in different cell lines without the need for the selection of single cell clones. The depletion of MSH6 was confirmed by immunoblot analysis of whole cell lysates and resistance to TMZ in a cell growth assay.

### 2.6. LN428 Cells Modified to Express MGMT

LN428 cells do not express MGMT due to promoter silencing [6]. We, therefore, modified LN428 cells to express MGMT by transfection with pIRES-Puro-MGMT and selection in puromycin, as previously described [6] (see the plasmid details in Supplementary Table S1).

### 2.7. Cell Growth Analysis

Cells (800) in growth medium (100 μL) were seeded in each well of a 96-well plate. The cells were then cultured overnight. On the second day, 200 μL of the treatment medium was added to each well after the removal of the overnight growth medium. Cells were then continuously cultured for 6–7 days. Subsequently, the total cell number following each treatment was determined using a Celigo Image Cytometer (Nexcelom Bioscience, Lawrence, MA, USA).

### 2.8. NAD^+^ Measurements

LN428/SCR or LN428/MSH6-KO cells (1.0 × 10^5^/well) were seeded in each well of a 96-well plate with 100 μL growth medium and cultured overnight. Then, the growth medium was removed and 100 μL NRH (100 μM)-containing medium was added at set time points. After a treatment of 1, 2, 4, 6, or 8 h with NRH, the medium was removed, and cells were kept at −80 °C until the subsequent NAD^+^ analysis experiment was performed. For the NAD^+^ assay, the cells in each well were washed with 1X PBS and then lysed for 5 min at room temperature with a 0.2 N NaOH (100 μL) solution supplemented with 1% dodecyl trimethylammonium bromide (DTAB), which preserves the stability of the dinucleotides. A Nanodrop Spectrophotometer (Thermo Fisher Scientific, Waltham, MA, USA) was used to determine the protein concentration for each sample (280 nm). Subsequently, the NAD^+^ concentration for each cell line was measured using the Promega NAD^+^/NADH-Glo Assay kit (Cat# G9071, Promega, Madison, WI, USA), as per the manufacturer’s instructions. For each sample, the lysate was diluted 1:50 in dilution buffer (equal volume of 0.2 N NaOH and 1X PBS). Subsequently, 50 μL of each sample was added in duplicate to the wells of a clear-bottom, white 96-well plate. HCl (0.4 N, 25 μL) was added to each well for the NAD^+^ measurements. Then, the plate was incubated at 60 °C for 15 min, followed by incubation at room temperature for 10 min. Tris-Cl, pH 10.7 (25 μL) was added to each well to neutralize the samples. The luminescence was recorded using a luminometer after adding 100 μL of the detection reagent to each well. For the generation of the NAD^+^ standard curve, NAD^+^ was diluted in dilution solution (1:1 volume of 0.2 N NaOH and 1× PBS or 1:1 volume of 0.4 N HCl and 1× PBS). The NAD^+^ concentration was calculated using the protein concentration of each sample for normalization.

### 2.9. Laser Micro-Irradiation

Laser micro-irradiation experiments were performed as we described previously [33], with slight modifications. First, LN248/MSH6-KD cells (5 × 10^4^ per well) were seeded into each well of an 8-chamber glass-bottom vessel (Thermo Fisher Scientific, Waltham, MA, USA, #155409) and cultured overnight. They were then modified to express EGFP-tagged XRCC1 (pLV-CMV-XRCC1-EGFP-Hygro; see Supplementary Table S1) by transfection using TransIT X2 according to the manufacturer’s instructions and cultured for 48 h. Next, a Nikon A1rsi confocal microscope was used for laser micro-irradiation. Cells were then imaged with a Nikon A1rsi laser scanning confocal microscope, which is equipped with a live-cell incubation chamber (Tokai Hit, Fujinomiya, Japan) maintained at 5% CO_2_ and 37 °C. A 20× (NA = 0.8) non-immersion objective was used for imaging. Micro-irradiation was performed using a 405-nm laser and the stimulation time was 0.125 s at the 100% power per site. Time lapse images were collected every 15 s during a 20-min interval. MIDAS software was used for the quantitation of the focal recruitment images and the statistical analysis of focal recruitment [33]. Recruitment profiles and kinetic parameters were generated from the analysis of at least 35 individual cells.

### 2.10. CometChip Analysis

DNA damage analysis by CometChip was performed as we described previously [34]. The 96-well CometChips, a well-former for the assembly, the CometChip Electrophoresis equipment (CES), and the Comet Analysis Software (CAS) were all purchased from BioTechne (Minneapolis, MN, USA). For acute DNA damage analysis by CometChip, cells were seeded into a 96-well plate (1 × 10^5^/well) and cultured overnight and then were pre-treated with DMSO, NRH, PARGi, or NRH plus PARGi for 4 h (doses indicated in the legends). Subsequently, the pre-treated cells were treated with TMZ or DMSO for an additional 1 h. Then, the cells were trypsinized and 150 µL cell-containing medium was transferred into each well of a CometChip. The CometChip assembly was kept at 4 °C for 30 min. Next, the CometChip was taken out from the CometChip assembly, washed twice with 30 mL 1× PBS, and sealed with 7 mL 0.75% low-melting-point agarose in 1× PBS (LMPA; Topvision; Thermo Fisher Scientific, Waltham, MA, USA). The cells in the CometChip were then lysed in lysis solution with detergent (BioTechne, Minneapolis, MN, USA) overnight at 4 °C. The CometChip was then electrophoresed in an alkaline solution (pH > 13; 200 mM NaOH, 1 mM EDTA, 0.1% Triton X-100) at 22 V for 50 min at 4 °C. Subsequently, the CometChip was neutralized to pH 7.4 by two washing steps with Tris buffer (0.4 M Tris·Cl, pH 7.4 and 20 mM Tris·Cl, pH 7.4). The DNA was then stained with 1× SYBR Gold dye (Thermo Fisher Scientific, Waltham, MA, USA) diluted in Tris buffer (20 mM Tris·Cl, pH 7.4) for 30 min. After the de-staining step (1 h) in Tris buffer (20 mM Tris·Cl, pH 7.4), the comet images were collected using a Celigo imaging cytometer (Nexcelom Bioscience; Lawrence, MA, USA) at a 1-μm/pixel resolution. The DNA damage (%Tail DNA) was quantified using the dedicated comet analysis software (CAS). The plots and statistical analyses were generated using Prism 9 (GraphPad Prism, San Diego, CA, USA). DNA damage is represented as % Tail DNA.

### 2.11. Statistical Analysis

For most analyses, data are shown as the mean ± standard deviation from 2 to 4 independent experiments. Student’s t-test was used when two groups were compared. One-way or two-way ANOVA was used for multiple comparisons. Statistical analysis was performed using Prism 9 (GraphPad Prism, San Diego, CA, USA).

## 3. Results

### 3.1. Loss of MSH6 in GBM Cells Results in TMZ Resistance That Can Be Overcome by Co-Treatment with NRH and PARGi

**Figure 1:**
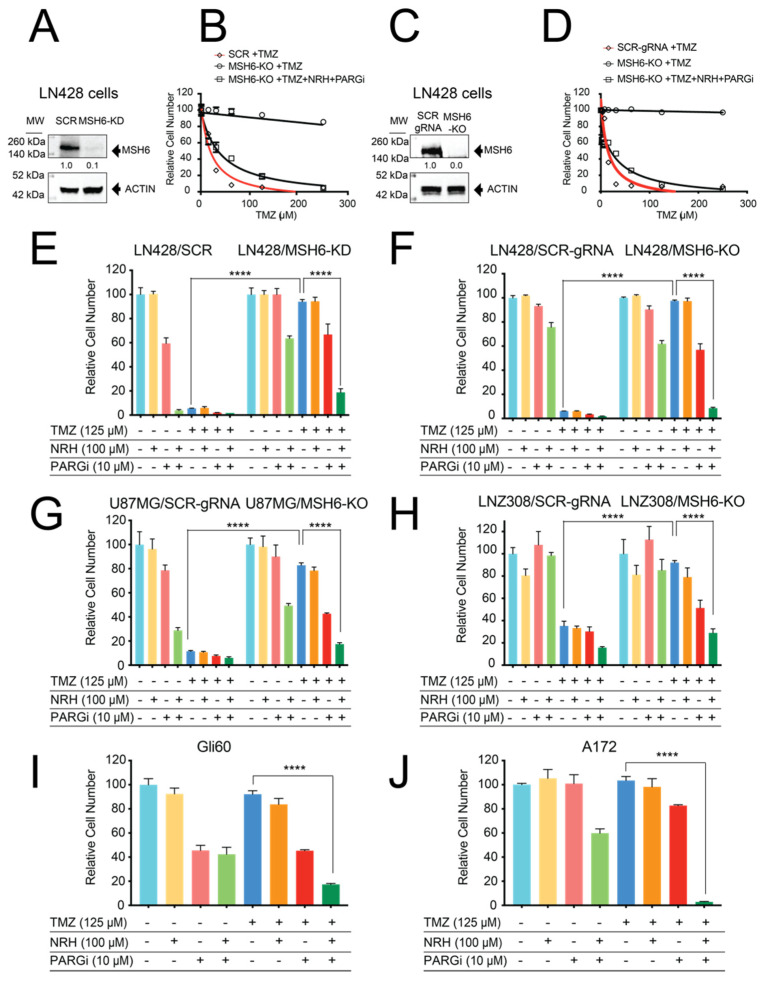
Loss of MSH6 in GBM cells resulted in TMZ resistance that was overcome by co-treatment with TMZ, NRH, and PARGi. (**A**) Immunoblot analysis of the MSH6 levels in LN428 cells transduced with lentivirus expressing shRNA-targeting MSH6 (KD) or a non-targeting scrambled shRNA (SCR). β-Actin was used as the loading control. (**B**) LN428/SCR or LN428/MSH6-KD cells after treatment with TMZ alone or TMZ supplemented with NRH (100 μM) + PARGi (10 μM) for 7 days (TMZ doses as indicated), normalized to DMSO. (**C**) Immunoblot analysis of the MSH6 levels in LN428 cells after MSH6 gene mutation (knockout, KO) by CRISPR/Cas9 with a pool of three gRNAs compared to a non-targeting scrambled gRNA (SCR-gRNA). β-Actin was used as the loading control. (**D**) LN428/SCR-gRNA or LN428/MSH6-KO cells after treatment with TMZ alone or TMZ supplemented with NRH (100 μM) + PARGi (10 μM) plus TMZ for 7 days (TMZ doses as indicated), normalized to DMSO. (**E**) LN428/SCR or LN428/MSH6-KD cells treated with DMSO, NRH (10 μM), PARGi (10 μM), N + P, TMZ (125 μM), T + N, T + P, or T + N + P for 7 days, normalized to DMSO: (**** *p* < 0.0001, two-way ANOVA). (**F**) LN428/SCR-gRNA or LN428/MSH6-KO cells treated with DMSO, NRH (100 μM), PARGi (10 μM), N + P, TMZ (125 μM), T + N, T + P, or T + N + P for 7 days, normalized to DMSO: (**** *p* < 0.0001, two-way ANOVA). (**G**) U87MG/SCR-gRNA or U87MG/MSH6-KO cells treated with DMSO, NRH (100 μM), PARGi (10 μM), N + P, TMZ (125 μM), T + N, T + P, or T + N + P for 7 days, normalized to DMSO: (**** *p* < 0.0001, two-way ANOVA). (**H**) LNZ308/SCR-gRNA or LNZ308/MSH6-KO cells treated with DMSO, NRH (100 μM), PARGi (10 μM), N + P, TMZ (125 μM), T + N, T + P, or T + N + P for 7 days, normalized to DMSO: (**** *p* < 0.0001, two-way ANOVA). (**I**) Gli60 cells (MSH6 mutant) treated with DMSO, NRH (100 μM), PARGi (10 μM), N + P, TMZ (125 μM), T + N, T + P, or T + N + P for 7 days, normalized to DMSO: (**** *p* < 0.0001, one-way ANOVA). (**J**) A172 cells treated with DMSO, NRH (100 μM), PARGi (10 μM), N + P, TMZ (125 μM), T + N, T + P, or T + N + P for 7 days, normalized to DMSO: (**** *p* < 0.0001, one-way ANOVA).

Loss of MSH6 expression in glioblastoma post treatment is one mechanism for acquired resistance to TMZ [18,35]. To evaluate the cellular phenotype of MSH6 loss and the resulting TMZ resistance in GBM cells, we reduced the expression of MSH6 (knockdown, KD) using shRNA (Figure 1A) or mutated the MSH6 gene (knockout, KO) using the CRISPR/Cas9 system (Figure 1C) in LN428 glioblastoma cells. As expected, both the resulting LN428/MSH6-KD and LN428/MSH6-KO cells were highly resistant to TMZ, with cell growth decreased by less than 10% at the highest TMZ concentration (250 µM), which is more than 2.5-fold higher than the clinical peak plasma concentration of TMZ (normally maximal at 100 µM) [36,37]. In contrast, MSH6-proficient control cells were highly sensitive to TMZ (Figure 1B,D). We previously reported that NRH treatment strongly potentiated the cytotoxicity of PARGi against glioma stem cells (GSCs) [25]. Therefore, we reasoned that the addition of both NRH (N) and PARGi (P) to the TMZ (T)-treated cells may overcome the resistance seen in the LN428/MSH6-KD and LN428/MSH6-KO cells [25]. As predicted, pre-treatment of the cells with NRH and PARGi, followed by TMZ treatment (T + N + P), restored the sensitivity to TMZ in both the LN428/MSH6-KD and LN428/MSH6-KO cells to that seen in the MSH6-proficient control cells (Figure 1B,D).

Then, we treated the MSH6-proficient or -deficient cells with TMZ at a lower dose, (closer to that used clinically, 125 µM) together with NRH (100 µM) or PARGi (10 µM), either alone or with different combinations. As shown (Figure 1E,F), MSH6-proficient control cells were sensitive to TMZ treatment alone or with any combined treatment with TMZ while MSH6-deficient cells were resistant to TMZ treatment alone. NRH treatment alone showed no inhibition of cell growth for either MSH6-proficient or -deficient LN428 cells. PARGi alone only induced minimal inhibition of cell growth in the LN428/MSH6-KD or LN428/MSH6-KO cells. However, for the combined treatments, **T**MZ plus **N**RH (T + N) treatment showed almost no inhibition of cell growth. The N + P treatment inhibited cell growth by less than 40% while **T**MZ plus **P**ARGi (T + P) treatment only reduced cell growth by less than 50% when treating the LN428/MSH6-KD or LN428/MSH6-KO cells. Only the co-treatment of T + N + P strongly inhibited the growth of LN428/MSH6-KD or LN428/MSH6-KO cells (Figure 1E,F).

To confirm that these results are applicable to different GBM cell lines, we mutated the MSH6 gene (knockout, KO) in U87MG and LNZ308 GBM cells using CRISPR/Cas9 and applied the same treatments to the control and resulting MSH6-KO cells. As seen in the LN428 cell line, the loss of MSH6 promoted TMZ resistance in both the U87MG and LNZ308 cell lines. TMZ resistance in both cell lines was only reversed by the T + N + P treatment regimen (Figure 1G,H). We next applied this treatment regimen to Gli60 cells, a GBM cell line encoding a null mutation in the MSH6 gene [18], and to A172 cells, a TMZ-resistant GBM cell line [38]. Both the Gli60 and A172 cells demonstrated a decrease in cell growth of less than 10% in response to TMZ treatment (125 µM) while the T + N + P treatment regimen strongly inhibited the cell growth of Gli60 cells (>80%) and fully inhibited the growth of A172 cells (Figure 1I,J). Altogether, our results demonstrate that the T + N + P treatment regimen overcomes TMZ resistance resulting from the loss of MSH6.

### 3.2. NRH Increased Cellular NAD^+^ Levels and Enhanced PARP1 Activity upon TMZ Treatment

**Figure 2:**
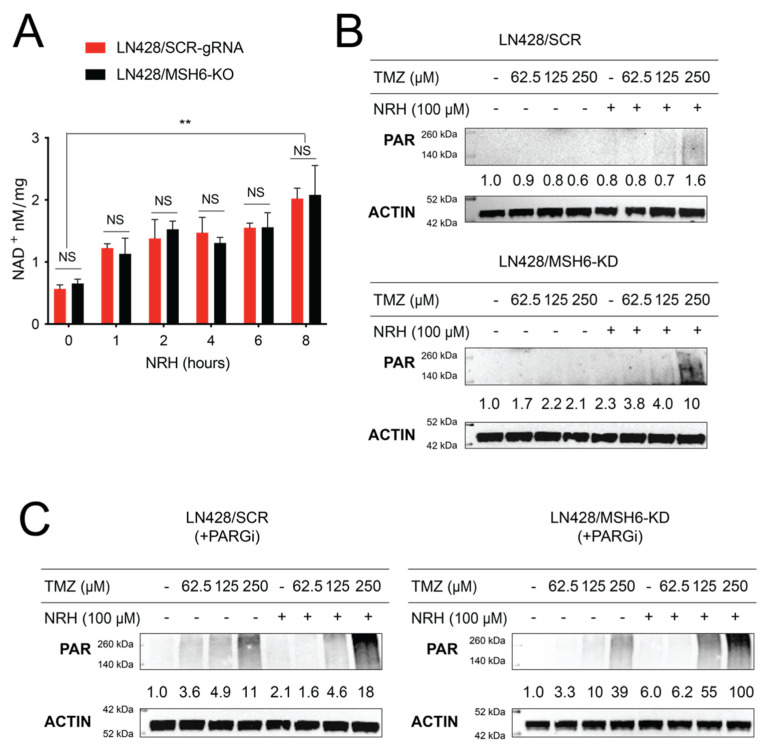
NRH treatment increased cellular NAD^+^ levels and promoted PARP1 activity upon TMZ treatment. (**A**) Total cellular NAD^+^ levels in LN428/SCR-gRNA or LN428/MSH6-KO cells after treatment with NRH (100 μM) for the time periods indicated; (** *p* < 0.01, one-way ANOVA; NS = not significant). (**B**) PAR (poly-ADP-ribose) immunoblot analysis of total cell lysates (LN428/SCR cells, top panel; LN428/MSH6-KD, bottom panel) after treatment with NRH (100 μM) for 4 h and then TMZ at the indicated doses for an additional 1 h, as compared to cells treated with DMSO. β-Actin was used as the loading control. (**C**) PAR (poly-ADP-ribose) immunoblot analysis of total cell lysates (LN428/SCR cells, left panel; LN428/MSH6-KD, right panel) after treatment with PARGi (100 μM) or N + P for 4 h and then TMZ at the indicated doses for an additional 1 h, as compared to cells treated with PARGi. β-Actin was used as the loading control.

To understand how the T + N + P treatment regimen overcomes TMZ resistance due to the loss of MSH6 expression, we first investigated how the increased bioavailability of NAD^+^ induced by NRH treatment impacts the BER pathway. NAD^+^ is a substrate for PARP1/PARP2, required for the generation of poly(ADP-ribose) (PAR) and is a regulatory factor for BER capacity [33,39,40]. Building on this BER regulatory role for NAD^+^, we reported that among a series of NAD-precursor molecules, nicotinomide riboside (NR), nicotinic acid riboside (NAR), dihydronicotinamide riboside (NRH), and dihydronicotinic acid riboside (NARH), only NRH was able to significantly increase the total cellular levels of NAD^+^ in non-stressed cells [25]. We then evaluated the cellular NAD^+^ levels of LN428/SCR-gRNA and LN428/MSH6-KO cells after NRH (100 µM) treatment. The total cellular NAD^+^ levels in both LN428/SCR-gRNA or LN428/MSH6-KO cells acutely increased after the addition of NRH (100 μM), reaching peak levels from 4 to 8 h (Figure 2A). There was no difference in the NAD^+^ level between LN428/SCR-gRNA and LN428/MSH6-KO cells, indicating MSH6 is not involved in this biosynthetic process, as expected [30]. Next, we evaluated the impact of increased cellular NAD^+^ levels on the activity of PARP1/PARP2 by immunoblotting analysis of PAR. Coincident with the increased NAD^+^ levels following NRH treatment, there was a significant increase in the level of PAR following TMZ treatment (Figure 2B). Further, we observed hyperaccumulation of PAR in both LN428/SCR and LN428/MSH6-KD cells following the T + N + P treatment regimen (Figure 2C).

### 3.3. NAD^+^ Bioavailability Modulated by NRH Together with PARGi Interferes with the Dynamics of BER Protein Complex Assembly/Disassembly and Inhibits the Repair of DNA Lesions from TMZ Treatment, Suppresses Survival Signaling, and Induces Apoptosis Signaling

**Figure 3:**
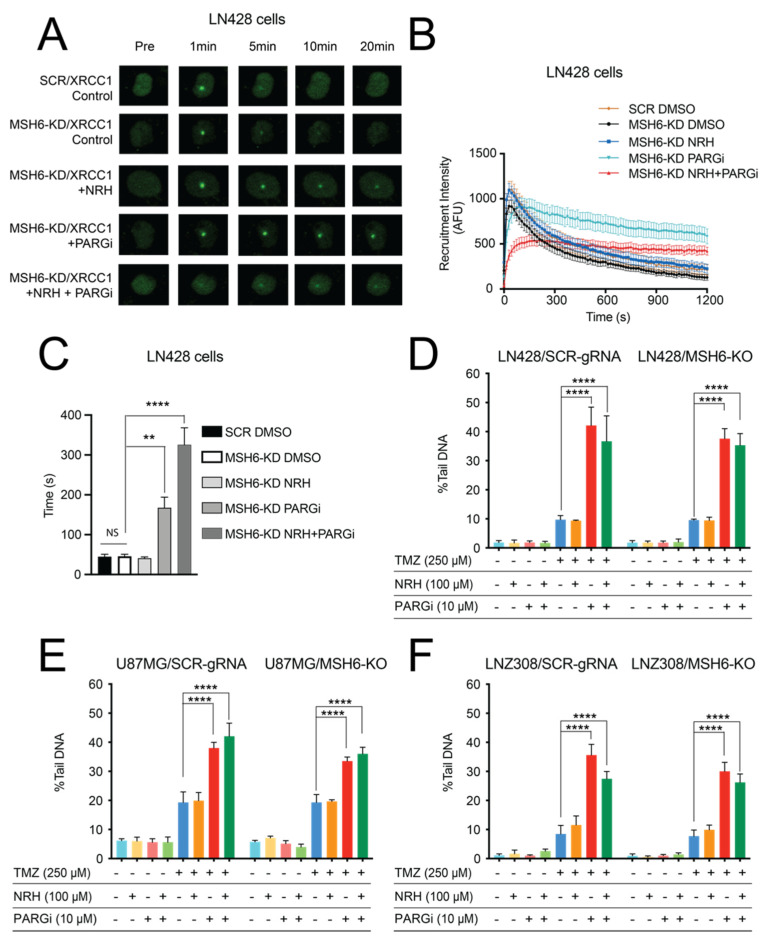
An NRH-mediated increase in NAD^+^ levels combined with PARGi interfered with the temporal dynamics of BER complex assembly/disassembly and blocked the repair of DNA lesions induced by TMZ treatment. (**A**) Representative images of XRCC1-EGFP expressed in LN248/MSH6-KD cells treated with DMSO, NRH (100 μM), PARGi (10 μM), or N + P for 4 h and then micro-irradiated. Foci in each image demonstrate XRCC1-EGFP recruitment to the site of laser-induced DNA damage. (**B**) Plot representing the recruitment kinetics of XRCC1-EGFP in LN428/SCR cells or in LN248/MSH6-KD cells treated with DMSO, NRH (100 μM), PARGi (10 μM), or N + P for 4 h and then micro-irradiated. (**C**) Time to the peak recruitment intensity of XRCC1-EGFP in LN428/SCR cells or in LN248/MSH6-KD cells treated with DMSO, NRH (100 μM), PARGi (10 μM), and N + P for 4 h and then micro-irradiated (NS = No significance, ** *p* < 0.01, **** *p* < 0.0001; one-way ANOVA). (**D**) LN428/SCR-gRNA and LN428/MSH6-KO cells were treated with DMSO, NRH (100 μM), PARGi (10 μM), or N + P for 4 h and then TMZ (250 μM) for an additional 1 h. DNA damage was evaluated by the CometChip assay, reported as % Tail DNA (**** *p* < 0.0001; two-way ANOVA). (**E**) U87MG/SCR-gRNA or U87MG/MSH6-KO cells were treated with DMSO, NRH (100 μM), PARGi (10 μM), or N + P for 4 h and then TMZ (250 μM) for an additional 1 h. DNA damage was evaluated by the CometChip assay, reported as % Tail DNA (**** *p* < 0.0001; two-way ANOVA). (**F**) LNZ308/SCR-gRNA or LNZ308/MSH6-KO cells were treated with DMSO, NRH (100 μM), PARGi (10 μM), or N + P for 4 h and then TMZ (250 μM) for an additional 1 h. DNA damage was evaluated by the CometChip assay, reported as % Tail DNA (**** *p* < 0.0001; two-way ANOVA).

**Figure 4:**
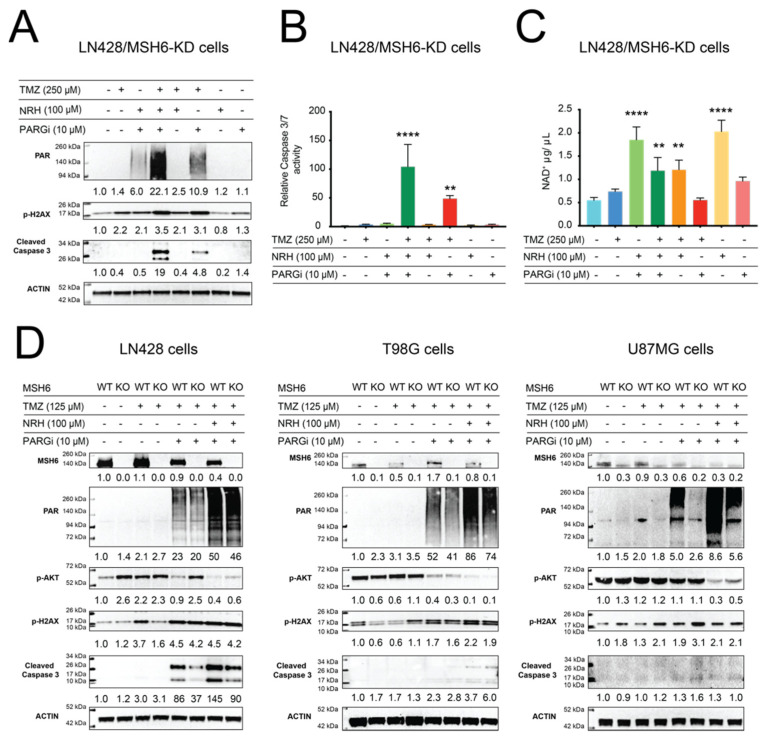
PAR accumulation suppresses cell survival signaling and induces apoptosis. (**A**) Immunoblot analysis of total cell lysates for PAR, γ-H2AX, and cleaved caspase 3 at 24 h after treatment of LN428/MSH6-KD cells with DMSO, NRH (100 μM), PARGi (10 μM), N + P, TMZ (250 μM), T + N, T + P, or T + N + P. β-Actin was used as the loading control. (**B**) Relative caspase 3/7 activity (LN428/MSH6-KD cells) 24 h after treatment with DMSO, NRH (100 μM), PARGi (10 μM), N + P, TMZ (250 μM), T + N, T + P, or T + N + P; normalized to DMSO (** *p* < 0.01, **** *p* < 0.0001, one-way ANOVA). (**C**) Total cellular NAD^+^ levels in LN428/MSH6-KD cells treated with DMSO, NRH (100 μM), PARGi (10 μM), N + P, TMZ (250 μM), T + N, T + P, or T + N + P for 24 h, normalized to DMSO (** *p* < 0.01, **** *p* < 0.0001, one-way ANOVA). (**D**) Immunoblot analysis of total cell lysates for MSH6, PAR, p-AKT, γ-H2AX, and cleaved caspase 3, 24 h after treatment of MSH6-WT or MSH6-KO from LN428, T98G, or U87MG cells treated with DMSO, TMZ (125 μM), T + P, or T + N + P. β-Actin was used as the loading control.

We next investigated how NRH + PARGi (N + P) treatment impacted the dynamics of BER protein complex assembly and disassembly. We used laser-induced micro-irradiation to induce DNA damage in cells and followed a fluorescently tagged XRCC1 transgene, a critical BER scaffold protein, as a biomarker for evaluating the dynamics of the BER complex [33]. LN428/SCR and LN428/MSH6-KD cells expressing XRCC1-EGFP were pre-treated with DMSO, NRH, PARGi, or NRH + PARGi for 4 h. The cells were then micro-irradiated (405-nm laser) and the recruitment of XRCC1-EGFP to the laser-induced DNA damage site was analyzed. XRCC1-EGFP was rapidly recruited to laser-induced DNA damage sites in both LN428/SCR and LN428/MSH6-KD cells when treated with DMSO (Figure 3). There was no significant difference in the recruitment dynamics (time to peak intensity) of XRCC1-EGFP between the LN428/SCR and LN428/MSH6-KD cells, indicating that MSH6 does not directly play a role in the BER pathway (Figure 3A–C). NRH treatment did not change the recruitment kinetics (the time to peak intensity) compared to the DMSO control while PARGi significantly increased the time to peak intensity. N + P treatment further increased the time to peak intensity (Figure 3B,C). Since XRCC1 recruitment is dependent on PAR [33], we reasoned that the delayed XRCC1-EGFP recruitment may relate to the change in the overall cellular PAR level after DNA damage in cells pre-treated with PARGi or N + P (Figure 2C and Figure 4A). Our previous report indicated that PARG inhibition resulted in elevated and persistent PAR at sites of laser-induced DNA damage that also delayed XRCC1 disassembly [33]. Similarly, there was a strong delay in the disassembly of XRCC1-EGFP from the laser-induced damage site after PARGi and almost no disassembly after N + P treatment (Figure 3A,B), indicating that PAR that accumulated at the laser-induced DNA damage site could not be degraded. 

PARGi or N + P treatment resulted in a defect in BER protein complex assembly and disassembly after laser-induced DNA damage. We hypothesized that the repair capacity of the BER pathway may also be impaired if PARGi or N + P treatment is administrated together with TMZ. To confirm this hypothesis, we evaluated the levels of DNA damage generated by treatment of cells with DMSO, NRH, PARGi, N + P, TMZ, T + N, T + P, or T + N + P using a 96-well CometChip platform [34]. There was a strong and acute increase in unrepaired DNA damage after T + P or T + N + P treatment when LN428, U87MG, or LNZ308 cells were exposed, regardless of MSH6 status (Figure 3D–F). There was no significant difference between the level of DNA damage induced by TMZ in MSH6-proficient or -deficient cells in all three cell lines (*p* > 0.9999 for each comparison of SCR-gRNA vs. MSH6-KO cells), suggesting that MSH6 is not directly involved in the repair of the TMZ-induced BER substrates N7-meG and N3-MeA, consistent with previous reports [8,9,14,16]. The increase in the level of unrepaired DNA damage in the T + P- or T + N + P-treated cells (24 h treatment) also induced the activation of the apoptosis pathway, as demonstrated by an increase in the level of cleaved caspase 3 (Figure 4A) and an increase in caspase 3/7 activity (Figure 4B). When measured 24 h after treatment, there was no loss in cellular NAD^+^ levels after TMZ plus PARGi treatment and an increase in cellular NAD^+^ levels after T + N + P treatment, compared to the DMSO control (Figure 4C). This excludes NAD^+^ depletion as the cause of cell death after PARP1 hyperactivation, as seen with TMZ treatment alone [32].

To understand what other factors may contribute to the inhibition of cell growth following the T + N + P treatment regimen, we examined signals for cell survival and cell death [41]. We next compared the activity of the survival signal AKT after treatment with TMZ, T + P, or T + N + P since nearly 90% of GBMs harbor activation of the AKT pathway [42]. As shown in Figure 4D, T + N + P treatment strongly suppressed the activation of AKT among the LN428, T98G, and U87MG cell lines regardless of MSH6 status. We also compared the death signal after 24 h, as indicated by cleaved caspase 3, from the same treatments. The level of cleaved caspase 3 induced by the T + N + P co-treatment regimen was more than that from the treatment of T + P in both the LN428 and T98G cells while TMZ treatment alone did not induce the cleavage of caspase 3. We did not detect cleaved caspase 3 in U87MG cells after treatment for 24 h, suggesting a longer treatment time may be needed (Figure 4A,B,D). Altogether, our results suggest that the T + N + P treatment regimen inhibits the BER pathway responsible for repairing a significant portion of TMZ-induced DNA damage, suppressed survival signaling, and induced apoptosis signaling after DNA damage induced by TMZ. As a result, the growth of the treated GBM cells was strongly inhibited.

### 3.4. Resistance to TMZ Treatment Due to MGMT Activity Was Overcome by Co-Treatment with TMZ, NRH, and PARGi

**Figure 5:**
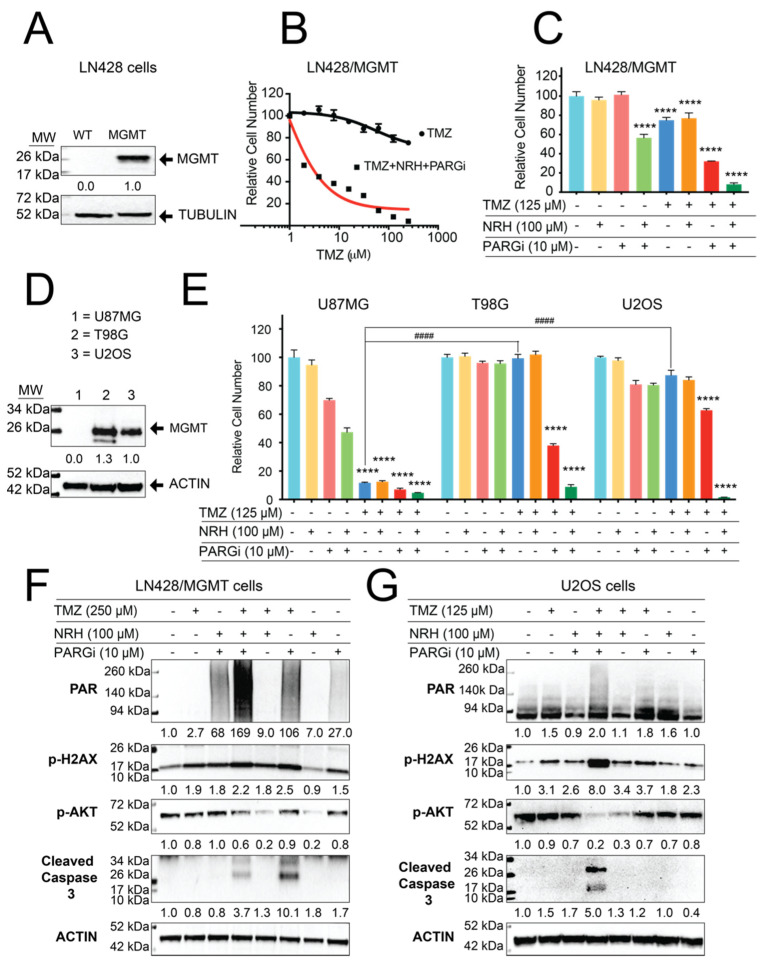
MGMT-mediated resistance to TMZ is overcome by a co-treatment with NRH and PARGi. (**A**) Immunoblot analysis of MGMT in LN428 cells (WT) modified to express human MGMT (LN428/MGMT). Tubulin was used as the loading control. (**B**) The relative number of LN428/MGMT cells after treatment with TMZ or NRH (100 μM) + PARGi (10 μM) plus TMZ for 7 days (TMZ doses as indicated), normalized to DMSO. (**C**) The relative number of LN428/MGMT cells after treatment with DMSO, NRH (100 μM), PARGi (10 μM), N + P, TMZ (125μM), T + N, T + P, or T + N + P for 7 days, normalized to DMSO (**** *p* < 0.0001, one-way ANOVA). (**D**) Immunoblot analysis of MGMT in U87MG, T98G, or U2OS cells. β-Actin was used as the loading control. (**E**) The relative number of U87MG, T98G, or U2OS cells after treatment with DMSO, NRH (100 μM), PARGi (10 μM), N + P, TMZ (125 μM), T + N, T + P, or T + N + P for 7 days, normalized to DMSO (**** *p* < 0.0001 for each DMSO control; ^####^ *p* < 0.0001 referred to TMZ treatment of U87MG cells, two-way ANOVA). (**F**) Immunoblot analysis of total cell lysates for PAR, p-AKT, γ-H2AX, and cleaved caspase 3, 24 h after treatment of LN428/MGMT cells with DMSO, NRH (100 μM), PARGi (10 μM), N + P, TMZ (250 μM), T + N, T + P, or T + N + P. β-Actin was used as the loading control. (**G**) Immunoblot analysis of total cell lysates for PAR, p-AKT, γ-H2AX, and cleaved caspase 3, after 24 h treatment of U2OS cells with DMSO, NRH (100 μM), PARGi (10 μM), N + P, TMZ (125 μM), T + N, T + P, or T + N + P. β-Actin was used as the loading control.

Another mechanism that promotes TMZ resistance is the expression of MGMT that directly repairs the O^6^-MeG lesion by transferring the methyl group to internal cysteine residues, thereby counteracting the cytotoxicity of TMZ [10]. Therefore, we further investigated the utility of the T + N + P treatment regimen in overcoming endogenous or acquired resistance to TMZ associated with MGMT activity. We overexpressed MGMT in LN428 cells (Figure 5A), in which endogenous MGMT expression is silenced by promoter methylation [6]. The LN428/MGMT cells were treated with TMZ or the T + N + P combination using different doses of TMZ. As expected, the elevated expression of MGMT in the LN428/MGMT cells conferred significant resistance to TMZ treatment. Cell growth of the LN428/MGMT cells was inhibited only by 25% when the cells were treated with high-dose TMZ (250 μM) while the T + N + P treatment regimen (at the same dose of TMZ) inhibited cell growth by 96% (Figure 5B). Similar to the MSH6-deficient cells, NRH or PARGi treatment alone did not show significant toxicity in the LN428/MGMT cells (*p* > 0.8) while N + P treatment inhibited cell growth by 44%. Co-treatment with T + P inhibited cell growth by 78% while co-treatment with T + N + P inhibited cell growth by 92% (Figure 5C).

To further validate our results, we applied this treatment regimen to the TMZ-resistant GBM cell line T98G, cells with endogenous elevated expression of MGMT, as compared to the TMZ-sensitive cell line U87MG, as a control (Figure 5D). TMZ (125 μM) treatment alone inhibited the growth of U87MG cells by 89% yet only inhibited the growth of T98G cells by 1%. However, co-treatment of T98G cells with the T + P regimen inhibited cell growth by 63% while co-treatment of T98G cells with the T + N + P combination inhibited cell growth by 91% (Figure 5E), using a TMZ dose of 125 μM.

To expand the utility of this T + N + P combination treatment regimen to other cancer cell types, we treated U2OS cells that have endogenous expression of MGMT and are resistant to TMZ treatment [43]. Here, TMZ (125 μM) inhibited the growth of U2OS cells by only 13%. However, co-treatment of U2OS cells with the T + P regimen inhibited growth by 47% while co-treatment with the T + N + P regimen completely inhibited U2OS cell growth (Figure 5E). Co-treatment of U2OS cells with T + N + P also resulted in hyperaccumulation of PAR and induced apoptosis and suppressed p-AKT levels (Figure 5F,G). This measure of anti-survival and pro-apoptotic signals at 24 h suggests that the cytotoxicity of the co-treatment of T + N + P is not associated with the O^6^-MeG lesion and is likely via failed BER-mediated repair and the onset of PAR-induced checkpoint activation [25]. Altogether, these results demonstrate that the T + N + P co-treatment regimen overcomes TMZ resistance from endogenous or acquired expression or function of MGMT.

## 4. Discussion

Over 80% of the DNA lesions (N7-MeG and N3-MeA) induced by TMZ are quickly repaired by the BER pathway [44]. Therefore, inhibition of the BER pathway offers a very promising target to overcome TMZ resistance that results from insensitivity to the O^6^MeG lesion (<10%) [45]. In the BER pathway, PARylation is a critical signal for the recruitment of the PAR-binding DNA repair factors to the proximity of DNA lesions [40,46], and dePARylation is needed to remove the linear or branched PAR chains for those repair factors to access DNA lesions [33]. Therefore, PARGi would result in an accumulation of PAR that will likely retain or trap BER factors at the site of DNA damage, thereby interrupting downstream repair [47,48]. Consistent with these reports, we demonstrated here that PARGi plus TMZ treatment induced a strong level of PAR accumulation while the addition of NRH (that modulated NAD^+^ bioavailability) further increased PAR hyperaccumulation (Figure 3, Figure 4 and Figure 5). Our micro-irradiation data shows that the kinetics of assembly and disassembly of BER factors (modeled by XRCC1) is interrupted by NRH + PARGi treatment (Figure 3). This interruption of DNA repair causes a strong increase in the level of unrepaired DNA damage in cells treated with TMZ plus PARGi or the T + N + P treatment regimen (Figure 3D–F), as compared to TMZ treatment alone. There is no significant difference in the level of unrepaired DNA damage when comparing cells treated with TMZ + PARGi vs. TMZ + NRH + PARGi (T + N + P), which may indicate that PARGi in both treatments fully blocked the capacity of BER to repair TMZ-induced DNA lesions. There is no difference in the level of DNA damage between the control (SCR-gRNA) and MSH6-KO cells (LN428, U87MG, or LNZ308 cell lines), indicating MSH6 is not immediately required to repair DNA lesions that arise following acute TMZ treatment. MMR plays a role only after the first replication past an unrepaired O^6^MeG lesion that gives rise to an O^6^Me-G/T mismatch [8,9]. TMZ plus PARGi or T + N + P induced apoptosis at 24 h (within the first cell cycle) after treatment, indicating that the cytotoxicity of the co-treatments is not related to the cytotoxicity of the O^6^MeG lesion in MSH6-deficient cells. The apoptosis signal that results from the cytotoxicity of the O^6^-MeG lesion only appears after the second cell cycle, with an intra-S-phase arrest after MNNG or TMZ treatment [9,49]. The apoptosis signal appeared at 24 h (first cell cycle) after co-treatment of T + N + P (Figure 5) in LN428/MGMT, U2OS (Figure 5), and T98G (Figure 4) cells, each harboring acquired or endogenous expression of MGMT, supporting our conclusion.

We observed that the GBM cells used in this study are not as sensitive to NRH + PARGi treatment as compared to GSCs [25]. NRH (100 μM) + PARGi (10 μM) treatment inhibited the cell growth of most of the GBM cell lines by less than 50% while there was more than 90% cytotoxicity of the GSCs with a much lower concentration of PARGi (~1 μM). This may result from the different molecular characteristics between GBM cells and glioma stem cells (GSCs). Firstly, GSCs achieved higher cellular NAD^+^ levels (10-fold) after treatment with NRH to promote PARP1 activity as compared to LN428 cells (4-fold) [25]. Secondly, GSCs only account for a small fraction of the total tumor cells with a significant increase in PARP1 expression as compared to the non-stem cell population [25,50]. Because the cytotoxicity of PARGi is largely dependent on the activity of PARP1 and higher PARP1 levels in GSCs mean stronger PARGi effects while NRH modulated NAD^+^ bioavailability further potentiates the cytotoxicity of PARGi. Thirdly, our previous report shows that the hyperaccumulation of PAR from NRH + PARGi-treated GSC cells is dependent on DNA replication and blocks replication fork progression [25]. It has been reported that CD133^+^ GSCs exhibited a reduced DNA replication velocity and a higher frequency of stalled replication forks than CD133^-^ non-GSC cells [51,52]. Using isogenic model cell lines, McGrail et al. revealed that cancer stem cells harbor defects in the replication stress response [53]. Therefore, the different sensitivity to NRH + PARGi treatment between GSCs and GBM cells may also relate to the replication status difference between the two cell types.

After DNA damage, the fate of cells between cell survival and cell death is decided by factors that are involved in DNA damage recognition, DNA repair, and DNA damage tolerance, and factors involved in the activation of apoptosis, necrosis, autophagy, and senescence [54]. It has been reported that the activity of AKT suppresses TMZ- or radiation-induced G2 arrest in cancer cells [55,56]. Inhibition of the activity of AKT by the PI3K inhibitor, LY294002, potentiated the cytotoxicity of TMZ against melanoma cell growth and invasion [57] and glioma cell growth [58]. However, the combination of TMZ and RAD001, a PI3K-AKT-mTOR inhibitor, failed in patients with metastatic melanoma in a phase II clinical trial, which might indicate other resistance mechanisms [59]. Consistent with this, our results showed even though the levels of p-AKT in TMZ-resistant cells (LN428/MGMT and U2OS) were strongly reduced when treated with TMZ plus NRH, the cell growth of both cell lines was only minimally reduced (Figure 4E–G). Even though there was no significant difference in the level of unrepaired DNA damage after the TMZ + PARGi or TMZ + NRH + PARGi (T + N + P) treatment regimens, the T + N + P treatment regimen resulted in a stronger accumulation of PAR, more suppressed survival signaling (p-AKT), and a higher level of the cell death signal (cleaved caspase 3). Therefore, the T + N + P treatment regimen inhibited the growth of all TMZ-resistant cells by more than 80% (Figure 1 and Figure 5). It was reported that PARP inhibition suppresses the PARylation of ATM, resulting in the translocation of an ATM-NEMO complex to the cytosol and interaction with mTOR to activate AKT for survival [60]. Therefore, it is conceivable that the T + N + P treatment regimen may retain PARylated ATM in the nucleus to reduce the activation of AKT after DNA damage. However, future experimental evidence is needed to validate this hypothesis. Additionally, the co-treatment of T + N + P strongly inhibited the cell growth of all TMZ-resistant cells, regardless of p53 status. Both LN428 and T98G cells express mutant p53 and LNZ308 does not express p53 while U87MG and U2OS express wild-type p53 [61,62]. Since the p53 pathway (including CDKN2A, MDM2, and TP53) is deregulated in ~85% of tumors [63], this p53-independent cytotoxicity from the co-treatment of T + N + P is very encouraging for GBM treatment.

## 5. Conclusions

**Figure 6:**
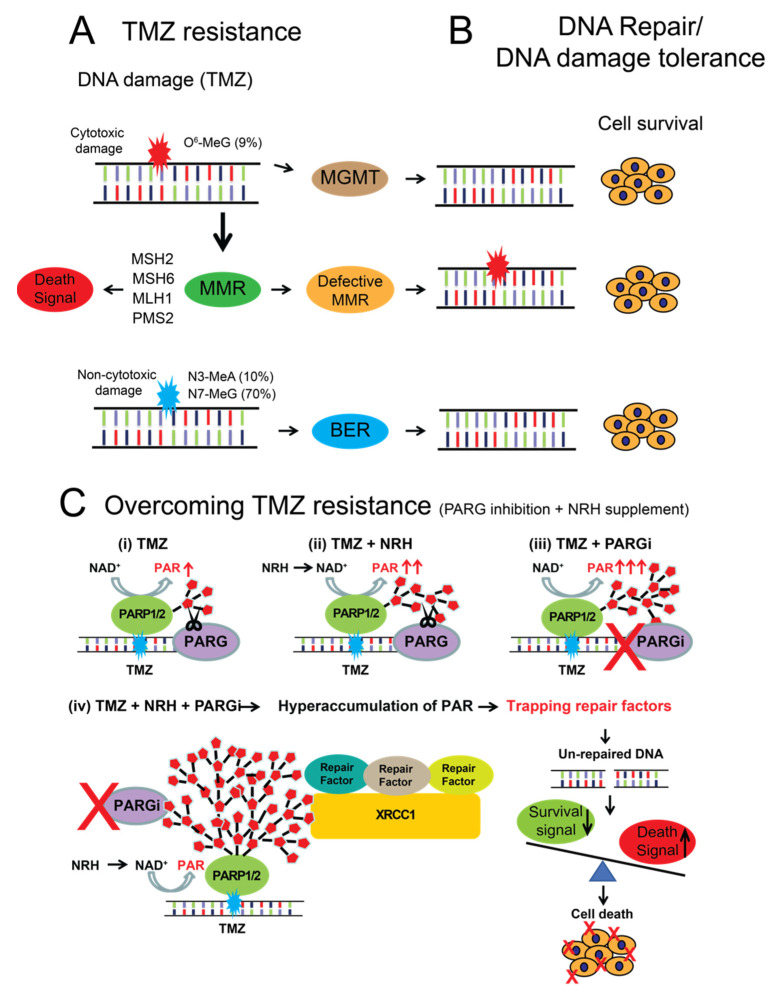
Proposed model of TMZ resistance mechanisms that can be overcome by co-treatment with NRH and PARGi. (**A**) TMZ generates multiple DNA lesions, including O^6^-methylguanine (O^6^-MeG, ~9%), N7-methylguanine (N7-MeG, >70%), and N3-methyladenine (N3-MeA, ~10%); (**B**) The O^6^-MeG DNA lesion can be directly repaired by MGMT leading to TMZ resistance. If the O^6^-MeG/T mis-pair is acted upon by proteins of the MMR pathway, this leads to cell death. However, when the MMR pathway is defective, due to loss of expression or mutations in genes of the MMR pathway, then the O^6^-MeG/T mis-pair is tolerated and leads to TMZ resistance. Finally, the N7-MeG or N3-MeA DNA lesions are repaired by proteins of the BER pathway; (**C**) (i) TMZ induces PARP1/PARP2 activation, inducing the synthesis of PAR; (ii) NRH increases NAD^+^, which enhances PARP1/PARP2 activation and promotes an increase in PAR formation; (iii) Inhibition of PARG blocks degradation of PAR; (iv) Co-treatment of TMZ, NRH, and PARGi results in the hyperaccumulation of PAR, trapping DNA repair factors and leading to an increase in unrepaired DNA damage, a decrease in cell survival signaling, and an increase in cell death signaling.

As summarized in Figure 6, this report demonstrates that enhancing tumor cell levels of NAD^+^ by the addition of NRH strongly increases the cytotoxicity of TMZ + PARGi co-treatment. Further, we found that the NRH + TMZ + PARGi co-treatment regimen overcame TMZ resistance due to either a deficiency in MSH6 or the activity of MGMT. NRH administration rapidly increases NAD^+^ levels in both TMZ-sensitive and -resistant GBM cells and enhances the accumulation of PAR following treatment with TMZ. Furthermore, NRH administration triggers hyperaccumulation of PAR following treatment with TMZ + PARGi. The NRH + PARGi treatment altered BER complex dynamics and impaired repair of the DNA damage induced by TMZ. The combination of NRH + TMZ + PARGi suppressed the survival signaling from p-AKT and activated apoptosis signaling. As a result, this treatment regimen strongly suppressed the cell growth of MSH6-depleted or MGMT-expressing GBM cells. Considering the lack of toxicity from NRH and the weak cytostatic effect of PARGi, this combination strongly improves the efficacy of TMZ to overcome treatment resistance.

## Figures and Tables

**Table 1:** Primary and secondary antibodies used in this study.

Target Antigen	Company	Catalogue #	Dilution
γ-H2AX	Cell Signaling Technology (Danvers, MA, USA)	9718s	1:1000
Phosphorylated Akt (Ser473)	Cell Signaling Technology (Danvers, MA, USA)	9271S	1:1000
β-Actin	Cell Signaling Technology (Danvers, MA, USA)	8457S	1:2000
MGMT	Cell Signaling Technology (Danvers, MA, USA)	58121S	1:1000
MSH6	Cell Signaling Technology (Danvers, MA, USA)	3995S	1:1000
Tubulin	Thermo Fisher Scientific (Waltham, MA, USA)	62204	1:1000
PAR	Gift from Mathias Ziegler(University of Bergen, Bergen, Norway)	N/A	1:1000
Cleaved Caspase 3	Cell Signaling Technology (Danvers, MA, USA)	9661S	1:1000
Immun-Star Goat anti-mouse-HRP conjugate	Bio-Rad (Hercules, CA, USA)	170-5047	1:5000
Immun-Star Goat anti-rabbit-HRP conjugate	Bio-Rad (Hercules, CA, USA)	170-5046	1:5000

## Data Availability

All data reported in this paper will be shared by the lead contact upon request. Any additional information required to reanalyze the data reported in this paper is available from the lead contact upon request.

## Supplementary Materials

The following supporting information can be downloaded at: https://www.mdpi.com/article/10.3390/cancers14153572/s1, Figure S1: Raw data of immunoblot from Figure 1A,C and Figure 2B,C; Figure S2: Raw data of immunoblot from Figure 4A,D; Figure S3: Raw data of immunoblot from Figure 5A,D,F,G; Table S1: Vectors developed for and used in this study.
