# Supplementary material for: Overcoming Temozolomide Resistance in Glioblastoma via Enhanced NAD+ Bioavailability and Inhibition of Poly-ADP-Ribose Glycohydrolase
Source: Cancers (Basel). 2022 Jul 22;14(15):3572. doi: 10.3390/cancers14153572 (PMC9331395; doi:10.3390/cancers14153572)
Supplement: Supplementary file 1 [file cancers-14-03572-s001.zip › cancers-1794690-supplementary.pdf]

Supplement for:

Overcoming temozolomide resistance in glioblastoma via enhanced NAD^+ bioavailability and inhibition of poly-ADP-ribose glycohydrolase

Jianfeng Li[1,2], Christopher A. Koczor[1,2], Kate M. Saville[1,2], Faisal Hayat[1,2], Alison Beiser[1,2], Steven McClellan[3], Marie E. Migaud[1,2] and Robert W. Sobol[1,2]*

[1] Department of Pharmacology, College of Medicine, University of South Alabama, Mobile, AL 36688, USA; jianfengli@southalabama.edu (J.L.); cakoczor@southalabama.edu (C.A.K.); mkm1325@jagmail.southalabama.edu (K.M.S.); fhayat@southalabama.edu (F.H.); avbeiser@southalabama.edu (A.B.); mmigaud@southalabama.edu (M.E.M.)
[2] Mitchell Cancer Institute, University of South Alabama, Mobile, AL 36604, USA
[3] Mitchell Cancer Institute Flow Cytometry SRL, University of South Alabama, Mobile, AL 36604, USA; smcclellan@health.southalabama.edu (S.M.)
* Correspondence: rwsobol@southalabama.edu (R.W.S.)

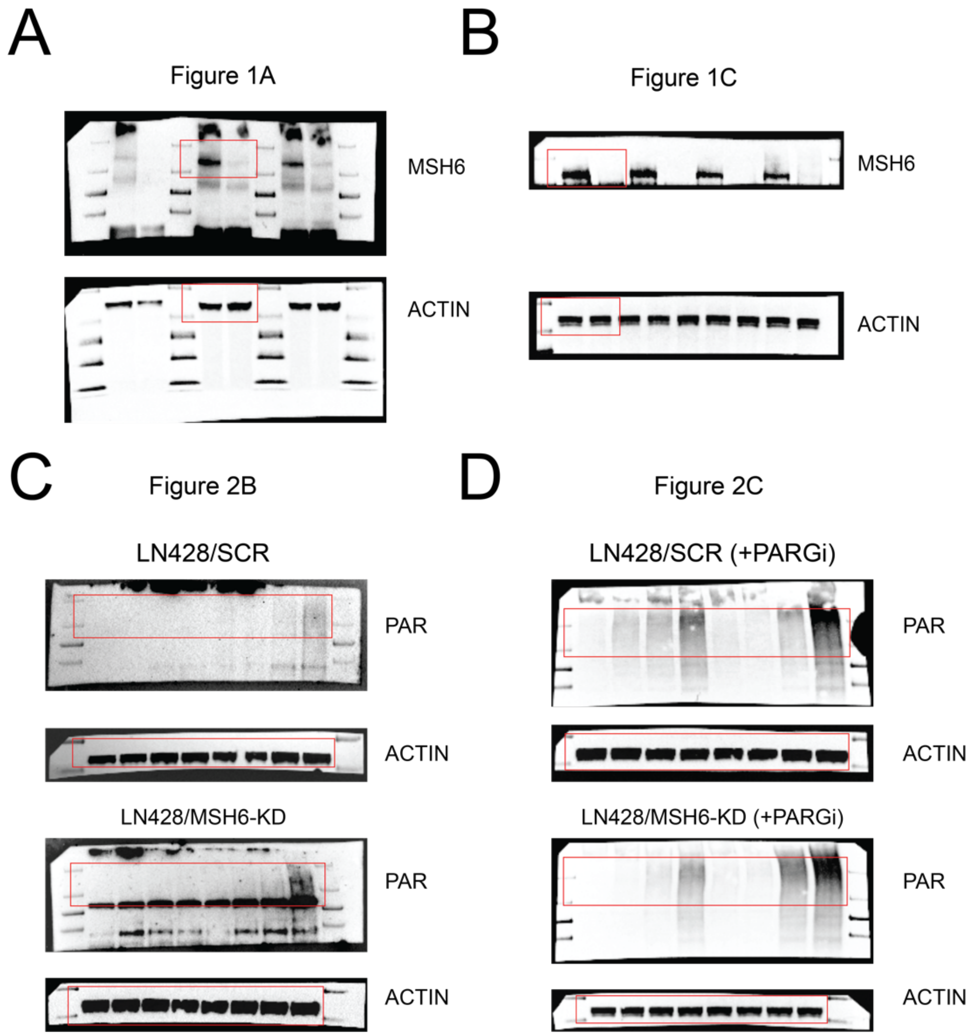

Figure S1. Raw data of immunoblot from Figure 1A, C and Figure 2B, C. (**A**) Raw data of immunoblot from Figure 1A. (**B**) Raw data of immunoblot from Figure 1C. (**C**) Raw data of immunoblot from Figure 2B. (**D**) Raw data of immunoblot from Figure 2C. The red rectangles represent the cropped images in each figure in the main body of the text.

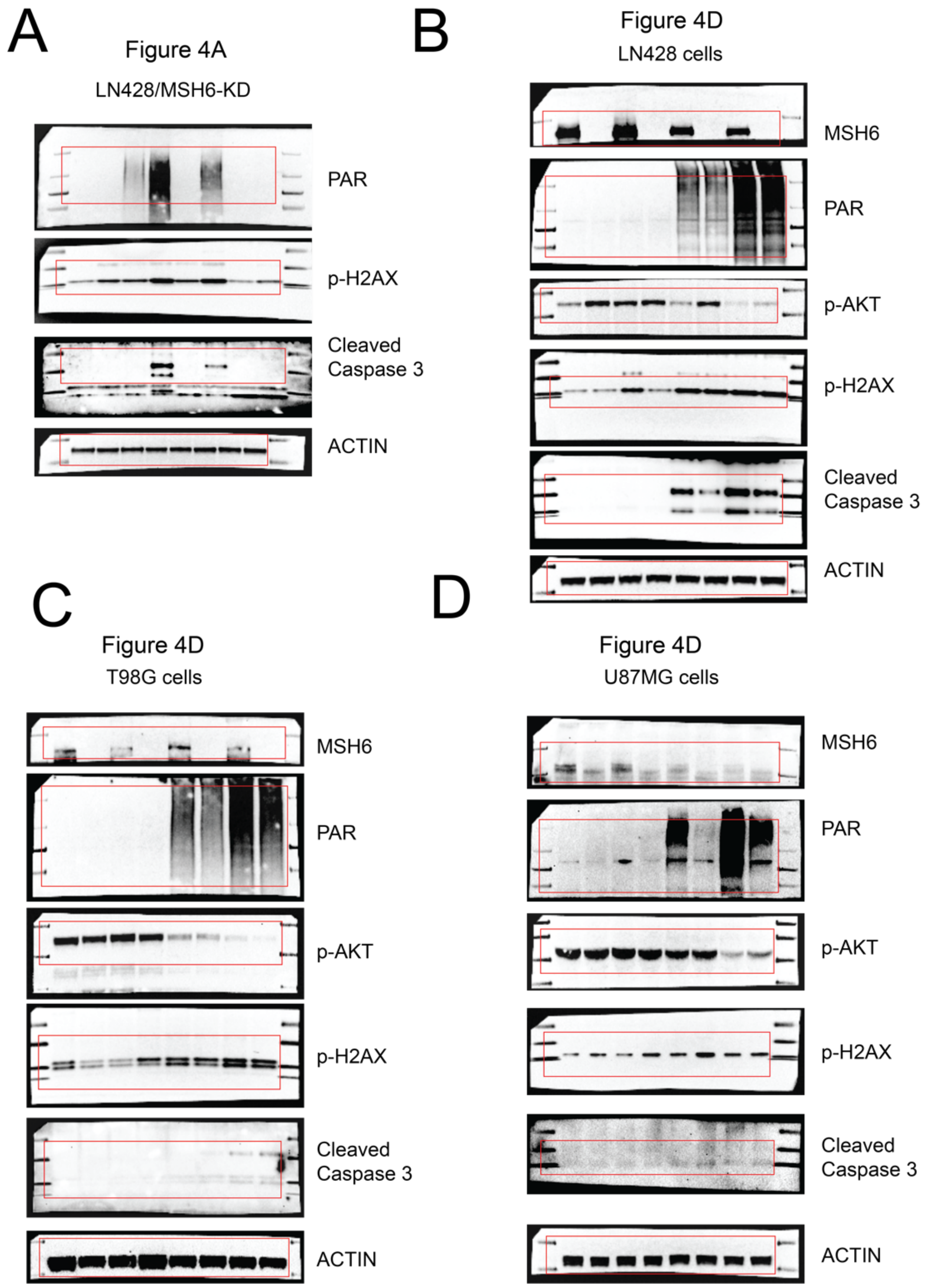

Figure S2. Raw data of immunoblot from Figure 4A, D. (**A**) Raw data of immunoblot from Figure 4A. (**B-D**) Raw data of immunoblot from Figure 4D. The red rectangles represent the cropped images in each figure in the main body of the text.

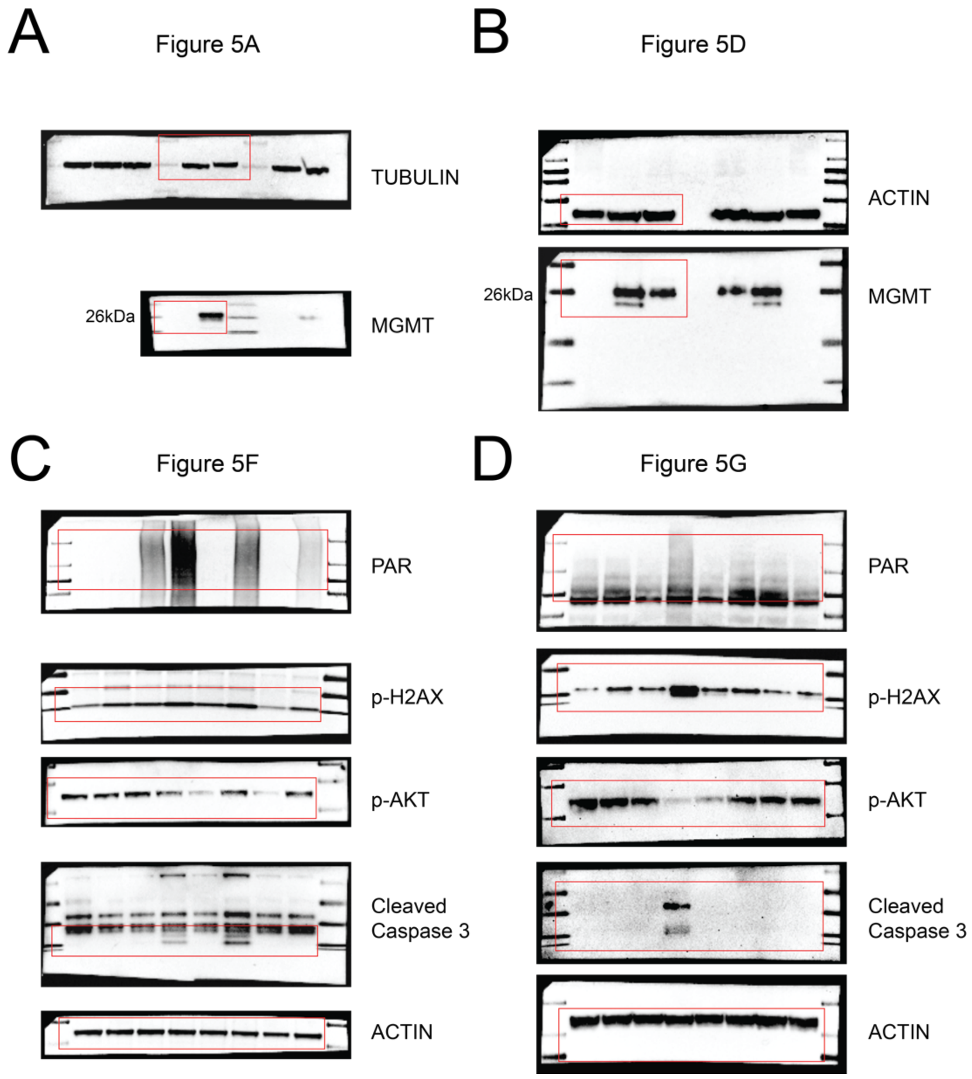

Figure S3. Raw data of immunoblot from Figure 5A, D, F, G. (**A**) Raw data of immunoblot from Figure 5A. (**B**) Raw data of immunoblot from Figure 5D. (**C**) Raw data of immunoblot from Figure 5F. (**D**) Raw data of immunoblot from Figure 5G. The red rectangles represent the cropped images in each figure in the main body of the text.

Supplementary Table S1. Vectors developed for and used in this study.

Lab Stock #	Plasmid name	Insert description	Availability
2323	pLenti-U6-sgRNA1(MSH6)-SFFV-Cas9-2A-Puro	MSH6 sgRNA #1 CRISPR/Cas9 All-in-One Lentivector set (Human)	ABM (Cat# 307361)
2324	pLenti-U6-sgRNA2(MSH6)-SFFV-Cas9-2A-Puro	MSH6 sgRNA #2 CRISPR/Cas9 All-in-One Lentivector set (Human)	ABM (Cat# 307361)
2325	pLenti-U6-sgRNA3(MSH6)-SFFV-Cas9-2A-Puro	MSH6 sgRNA #3 CRISPR/Cas9 All-in-One Lentivector set (Human)	ABM (Cat# 307361)
1800	pLenti-CRISPR-cas9-Scramble	Scramble sgRNA CRISPR/Cas9 All-in-One Lentivector (control)	ABM (Cat# 307361)
1726	pLV-CMV-XRCC1-EGFP-Hygro	EGFP fused to the C-terminus of XRCC1 & a hygromycin resistance cassette.	Addgene #176062
252	pMD2.g(VSVG)	VSV-G; HIV lentiviral packaging plasmid	Addgene #12259
253	pVSV-REV	Rev; HIV lentiviral packaging plasmid	Addgene #12253
254	pMDLg/pRRE	gag and pol; HIV lentiviral packaging plasmid	Addgene #12251
665	pLKO.1-puro-shSCR	Scrambled shRNA	Millipore-Sigma (SHC002)
sh25.1	pLKO.1-puro-shMSH6.1	shRNA targeting Human MSH6 gene	Millipore-Sigma (TRCN0000078543; target seq = GCCAGAAGAATACGAGTTGAA)
422	pIRES-Puro-MGMT	hMGMT	Addgene # 187654
